# Chikungunya: A Potentially Emerging Epidemic?

**DOI:** 10.1371/journal.pntd.0000623

**Published:** 2010-04-27

**Authors:** Michelle M. Thiboutot, Senthil Kannan, Omkar U. Kawalekar, Devon J. Shedlock, Amir S. Khan, Gopalsamy Sarangan, Padma Srikanth, David B. Weiner, Karuppiah Muthumani

**Affiliations:** 1 Department of Earth and Environmental Sciences, University of Pennsylvania School of Medicine, Philadelphia, Pennsylvania, United States of America; 2 Department of Pathology and Laboratory Medicine, University of Pennsylvania School of Medicine, Philadelphia, Pennsylvania, United States of America; 3 Inovio Biomedical Corporations, Blue Bell, Pennsylvania, United States of America; 4 Department of Microbiology, Sri Ramachandra Medical Centre, Sri Ramachandra University, Tamil Nadu, India; London School of Hygiene & Tropical Medicine, United Kingdom

## Abstract

Chikungunya virus is a mosquito-borne emerging pathogen that has a major health impact in humans and causes fever disease, headache, rash, nausea, vomiting, myalgia, and arthralgia. Indigenous to tropical Africa, recent large outbreaks have been reported in parts of South East Asia and several of its neighboring islands in 2005–07 and in Europe in 2007. Furthermore, positive cases have been confirmed in the United States in travelers returning from known outbreak areas. Currently, there is no vaccine or antiviral treatment. With the threat of an emerging global pandemic, the peculiar problems associated with the more immediate and seasonal epidemics warrant the development of an effective vaccine. In this review, we summarize the evidence supporting these concepts.

## Introduction

**Figure 1:**
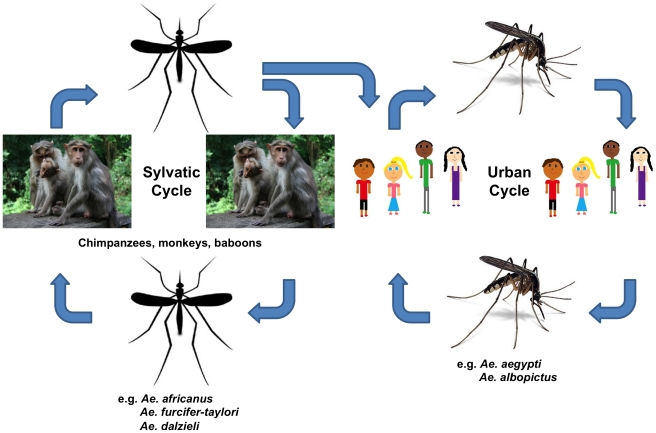
Life cycle of Chikungunya virus in Africa showing the interconnection between the sylvatic cycle on the left and the urban cycle on the right. Particularly in Africa, the virus is maintained in a sylvatic cycle comprising non-human primates and different species of forest-dwelling mosquitoes including *Aedene* mosquitoes (*Ae. Africanus*, *Ae. furcifer-taylori*, *Ae. dalzieli*, etc.,) and non *Aedene* mosquitoes (Mansonia, Culex, etc.) [10].

Chikungunya virus (CHIKV), a mosquito-borne pathogen listed by National Institute of Allergy and Infectious Diseases (NIAID) as a Category C Priority Pathogen that causes Chikungunya fever (CHIKF), has been spreading throughout Asia, Africa, and parts of Europe in recent times [1], [2], [3]. CHIKV is an arthropod-borne virus (arbovirus) and is transmitted to humans primarily by *Aedes aegypti*, the infamous yellow fever propagator [4], [5]. CHIKV infection is marked by severe joint pain, contorting its victims into unusual postures [6]. The disease gets its name from the Kimakonde vernacular language of Tanzania and Mozambique, and the word chikungunya means “that which contorts or bends up” and translates in Swahili to “the illness of the bended walker” [7],[8],[9]. In Africa, CHIKV is maintained in a sylvatic cycle among forest-dwelling *Aedes spp.* mosquitoes, wild primates, squirrels, birds, and rodents (Figure 1) [10]. In Asia, the disease is vectored by *Ae. aegypti* and *Ae. albopictus* [11]. Transmission in Asia occurs in an urban cycle whereby the mosquito spreads the disease from an infected human to an uninfected human, following an epidemiological pattern similar to dengue fever [12].

The 2005–2006 epidemic of CHIKV in La Reunion islands in the Indian Ocean, spurred the discovery of a new vector species, *Ae. albopictus* [5]. Wrecking over one-third of the island's population, this epidemic peaked its devastation between January and February 2006, when over 46,000 cases came into light every week, including 284 deaths [5], [13]. *Ae. albopictus* is common in urban areas of the United States and is already flourishing in 36 states, raising grave concerns to the immunologically naive populace of the United States [14].

Accordingly, this review elaborately details the epidemiology and global expansion of CHIKV, describes its clinical features and pathogenesis and its symptoms and complications, and finally nominates a possible vaccine approach against CHIKV infection.

## CHIKV Emergence

CHIKV has been isolated into three genotypes based on phylogenetic studies. These genotypes, based on the gene sequences of an Envelope protein (E1), are Asian, East/Central/South African, and West African [4], [11], [15]. Using phylogenetic models, Cherian et al. estimate that the Asian genotype of CHIKV emerged between 50 and 310 y ago, and the West and East African genotypes diverged between 100 and 840 y ago [15]. Since then, CHIKV has come a long way, with several mutations incorporated, and has continued to wreak epidemics in several regions. Recent activities of CHIKV include the Indian epidemic in 2005–2006, which was followed by a sudden explosion of cases in 2007. An estimated 1.3 million people across 13 states were reported to be infected in India [12], [16], and CHIKV was also widespread in Malaysia, Sri Lanka, and Indonesia [17]. In July–August of 2007, CHIKV was reported in Italy, probably brought in by travelers from CHIKV-prone regions of India, Africa, and Indian Ocean islands such as Mauritius, Madagascar, and Seychelles. Few of the Italian isolates were found to have evolved from the Kerala isolate, which was associated with a A226V shift in E1 gene that represents a successful evolutionary adaptation in the mosquito vector similar to the ones observed in Reunion Island [2], [18], [19].

In recent times, with an increase in global travel, the risk for spreading CHIKV to non-endemic regions has heightened [1]. Several travelers have brought CHIKV home with them after visiting areas with actively infected populations [12], [20]. Such cases have been documented in European countries, Australia, Asia, and the United States [8], [21]. The United States has already reported at least twelve cases of travel-associated CHIKV, while France has reported 850 cases, and the United Kingdom 93 [8], [14]. Beyond this, CHIKV-infected travelers have also been diagnosed in Australia, Belgium, Canada, Czech Republic, French Guiana, Germany, Hong Kong, Italy, Japan, Kenya, Malaysia, Martinique, Norway, Switzerland, and Sri Lanka [21]. Some travelers were viremic, worrying public health officials about the spread of CHIKV to new areas [1], [8].

## Symptoms and Complications

The incubation time for CHIKV is relatively short, requiring only 2–6 d with symptoms usually appearing 4–7 d post-infection [22]. Vazeille et al. detected CHIKV in the salivary glands of *Ae. albopictus* only 2 d after infection [5]. Upon infection, CHIKF tends to present itself in two phases. The first stage is acute, while the second stage, experienced by most but not all, is persistent, causing disabling polyarthritis. Characteristics of the acute phase include an abrupt onset of fever, arthralgia, and in some cases, maculopapular rash [6], [23]. The acute phase causes such intense joint and muscular pain that makes movement very difficult and prostrates its victims [6], [20].

Ninety-five percent of infected adults are symptomatic after infection, and of these, most become disabled for weeks to months as a result of decreased dexterity, loss of mobility, and delayed reaction. Eighteen months after disease onset, 40% of patients are found to still have anti-CHIKV IgM [6], [18], [23], [24]. The chronic stage of CHIKF is characterized by polyarthralgia that can last from weeks to years beyond the acute stage [6]. CHIKV has been shown to attack fibroblasts, explaining the involvement of muscles, joints, and skin connective tissues. The high number of nociceptive nerve endings found within the joints and muscle connective tissues can explain pain associated with CHIKF [25], [26].

More than 50% of patients who suffer from severe CHIKF are over 65 y old, and more than 33% of them die. Most adults who suffer from severe CHIKF have underlying medical conditions [6], [24], [27]. The other group that is disproportionately affected by severe CHIKV is children. Other complications associated with CHIKV, from most common to least common, include respiratory failure, cardiovascular decompensation, meningoencephalitis, severe acute hepatitis, severe cutaneous effects, other central nervous system problems, and kidney failure [6], [18], [20], [23], [24], [26], [27].

## CHIKV Viral Mutation and Resulting Increase in Epidemic Potential

**Figure 2:**
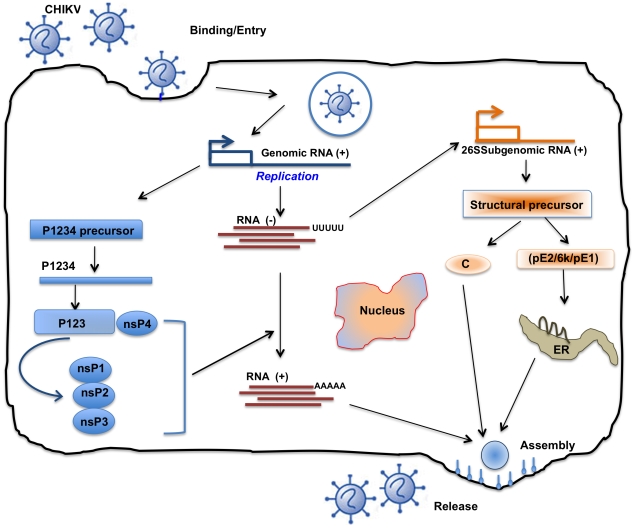
Life cycle of Chikungunya virus inside infected cells. Characteristically, there are two rounds of translation: (+) sense genomic RNA (49S′ = 11.7 kb) acts directly as mRNA and is partially translated (5′ end) to produce non-structural proteins (nsp's). These proteins are responsible for replication and formation of a complementary (−) strand, the template for further (+) strand synthesis. Subgenomic mRNA (26 S = 4.1 kb) replication occurs through the synthesis of full-length (−) intermediate RNA, which is regulated by nsp4 and p123 precursor in early infection and later by mature nsp's. Translation of the newly synthesized sub-genomic RNA results in production of structural proteins such as Capsid and protein E2-6k-E1 (from 3′ end of genome). Assembly occurs at the cell surface, and the envelope is acquired as the virus buds from the cell and release and maturation almost simultaneous occurred. Replication occurs in the cytoplasm and is very rapid (∼4 h) [28], [29].

CHIKV undertakes a complex replication cycle upon host infection (Figure 2), which makes its genome susceptible to mutations [28], [29]. For instance, *Ae. aegypti*, responsible for epidemics in Kenya, Comoros, and Seychelles, carried CHIKV with an alanine in the 226 position of the E1 gene (E1-A226) [4], [18]. However, when the virus struck La Reunion Islands, a decline in population of *Ae. aegypti*, due to massive dichlorodiphenyltrichloroethane usage and dearth of *Ae. albopictus* species' population, resulted in an ecological pressure, favoring replacement of alanine at position 226 with valine (E1-A226V) [5]. This mutation allowed CHIKV's secondary vector species, *Ae. albopictus*, to supplement *Ae. aegypti* as its primary vector [5].

Within a year, the E1-A226V mutation was present in La Reunion Island, and *Ae. albopictus* apparently vectored the large epidemic infecting 34% of La Reunion Island's population [5]. All of the CHIKV strains isolated from Mayotte carried the E1-A226V mutation, and the mutation was also found in Madagascar in 2007 [5]. The E1-A226V mutation was not present at the beginning of the Indian Ocean Islands outbreak (before September 2005). However, more than 90% of later viral strains found there had incorporated the mutation (December–March 2006), indicating a genotype switch during the winter season [5], [18], [20].

The E1-A226V mutation also enabled an increase in infectivity of *Ae. albopictus* when compared to its infectivity of *Ae. aegypti* [4], [11], [18], [30], and with several factors taken together, *Ae. albopictus* has become the new preferred and more lethal vector for CHIKV [4], [5], [11]. In fact, Tsetsarkin et al. found that a Green Fluorescent Protein tagged E1-A226V virus was 100 times more infective to *Ae. albopictus* than it was to *Ae. aegypti* [4]. In all the Indian Ocean Islands, *Ae. albopictus* became the main vector for CHIKV within 1–2 y after CHIKV was introduced to the region [31].

Of note is that *Ae. aegypti* has most likely been established in North America for over 300 y, while *Ae. albopictus* has been in many areas of the US, since 1985, primarily in Florida [32] and since then has expanded its range in the country. Reiskind et al. set out to determine if *Ae. aegypti* and *Ae. albopictus* mosquitoes captured in Florida were susceptible to CHIKV infection by a La Reunion isolate [32]. Each mosquito tested was highly susceptible to infection by a full-length infectious clone of the La Réunion Island isolate, CHIKV LR2006 OPY1 strain. Even though the *Ae. albopictus* strains were more susceptible to infection, overall ecology and differences in human biting patterns need to be studied further to gain a more accurate understanding of a potential CHIKV epidemic in the US [32].

## Vertical Transmission

During the 7 d preceding birth, no human mother has been reported to transmit the disease vertically. However, about 50% of newborns delivered while the mother was infected with CHIKV contracted the disease from their mother, despite the method of delivery. Furthermore, there have been instances of CHIKV transmission from mother to fetus causing congenital illness and fetal death [33].

During the 2005–2006 La Reunion Island outbreaks, Ramful et al. discovered that mothers could transmit CHIKV to their progeny during the perinatal period (Day −4 to Day +1) [33], [34], and it is associated with a high degree of morbidity. By mean Day 4 of life, all of the neonates were symptomatic for CHIKV, exhibiting common CHIKF symptoms. Six neonates were confirmed to have contracted CHIKV and developed mengoencephalitis. Of those mothers who, during the La Reunion Island epidemic, were infected long before delivery, only three fetal deaths were reported [12], [33]. Ramful et al. theorized that mother-to-child transmission most likely happens transplacentally shortly before delivery [33]. A similar study by Gerardin et al. reported nineteen cases of neonatal infection associated with intrapartum maternal viremia that progressed to develop encephalitis owing to vertical transmission from infected mothers [34].

## CHIKV Diagnosis

Clinical and epidemiological similarities with dengue fever make CHIKV diagnosis difficult, which may lead physicians to misdiagnose CHIKV as dengue fever; therefore, the incidence of CHIKV may actually be higher than currently believed (Table 1) [6], [12], [35].

**Table 1:** Comparison of clinical features of Chikungunya and Dengue virus.

Clinical Features	Chikungunya Virus (CHIKV)	Dengue Virus (DENV)	Reference
1) Fever, asthenia	Common	Common	[6], [8]
2) Myalgia	Possible	Very common	[6]
3) Polyarthritis	Very Common, edematous	None	[56]
4) Tenosynovitis	Yes	None	[57]
5) Leukopenia	None	Yes	[58]
6) Thrombocytopaenia	None	Yes	[59]
7) Rash	Days 1–4, important skin edema	Days 3–7	[6], [35], [58]
8) Retro-orbital pain	Rare	Common	[60]
9) Hypotension	Possible	Common, Days 5–7	[60], [61]
10) Minor bleeding	Chronic polyarthritis up to 1 year	Common	[17], [56]
11) Second stage	Possible; Tenosynvovitis at M2–M3 Raynaud's syndrome at M2–M3	Fatigue up to 3 mo	[6], [56], [57], [58], [62], [63]

The amount of time elapsed since disease onset is the most critical parameter when choosing a diagnostic test. CHIKV can be detected and isolated by culturing with mosquito cells (C6/36), Vero cells (mammalian), or in mice [26]. However, this method can take at least a week and only achieves a high sensitivity during the viremic phase, which usually only lasts up to 48 h after the bite. Five days post-infection, the viral isolation approach has a low sensitivity but is still the preferred method for detecting the CHIKV strain [12], [26], [31], [35]. RT-PCR on the other hand is a faster and more sensitive method that can be used within the first week of disease onset [26], and it is currently the most sensitive method for detecting and quantifying viral mRNA [4], [36].

Classic serological detection, by assays such as ELISA [37], immunofluorescence [5], [38], complement binding, and haemagglutination inhibition [39], constitutes the second diagnostic tool used for biological diagnosis of CHIKV infection. These proven techniques are useful for detection of Antigen in mosquitoes during epidemiological studies. These assays detect virus-specific IgM and IgG, however the sensitivity and specificity of these assays has been poorly characterized. Viral competence, or the potential of viral infection and transmission, is an important parameter that can be quantified by ELISA, viral culture, and PCR.

A study by Ng et al. showed biomarkers indicative of severe CHIKV infection [40]. They found decreased levels of RANTES and increased levels of Interleukin-6 (IL-6) and Interleukin-1β (IL-1β) that could be sued for CHIKV detection in patients as indicators of CHIKV-driven cytokine storm. Couderc et al. demonstrate another cytokine, type-I IFN, as a key player in the progression to CHIKV infection [26]. Using an IFN-α/β null mouse model, they demonstrated evidence of muscles, joints, and skin as privileged CHIKV targets, which is consistent with human pathology. Although Ng et al. concluded that RANTES levels were significantly suppressed in severe CHIKF patients [40], interestingly, an increase in levels of RANTES has been observed in dengue infection [41]. Since the symptoms of CHIKF mimic those of dengue fever, results obtained from this study strongly suggest that RANTES could be a potential distinctive biomarker that differentiates between these two clinically similar diseases.

## Vaccine Approach against CHIKV Infection

There are no approved antiviral treatments currently available for CHIKV [1], [3], [12], [42]. Currently, CHIKF is treated symptomatically, usually with non-steroidal anti-inflammatory drugs or steroids, bed rest, and fluids. Movement and mild exercise are thought to decrease stiffness and morning arthralgia, but heavy exercise may exacerbate rheumatic symptoms. Corticosteroids may be used in cases of debilitating chronic CHIKV infection. There is a debate about the appropriateness of chloroquine as treatment for unresolved, non-steroidal anti-inflammatory drug-resistant arthritis [43]. A study showed that viral production was drastically reduced at 16 h post-infection after treatment with 100 mM dec-RVKR-cmk (Decanoyl-Arg-Val-Lys-Arg-chloromethylketone), a furine inhibitor [42], [44]. Chloroquine acted by raising the pH, blocking low pH-dependent entry of virus into the cell. It is important to note that dec-RVKR-cmk or chloroquine only inhibited viral spreading from cell to cell, not CHIKV replication once it had entered the cell [43].

However, most would agree that the best weapon against CHIKV is prevention. A live CHIKV vaccine developed by the United States reached phase II clinical trial encompassing 59 healthy volunteers [45]. Eight percent of the volunteers experienced transient arthralgia, while 98% of the volunteers had seroconversion [45]. However, live CHIKV vaccines are still questionable. One cannot discount the risk of a live vaccine possibly inducing chronic rheumatism. Also, there is the question as to whether widespread use among the public could trigger mosquito transmission or lead to chronic infection or viral reversion [1].

An alternative approach would be to produce a chimeric vaccine against CHIKV. Wang et al. developed a chimeric alphavirus vaccine that is uniformly attenuated and does not cause reactogenicity in mice [3]. Three different versions of this vaccine were made using three different backbone vectors: Venezuelan equine encephalitis virus (VEEV) attenuated vaccine strain T-83, naturally attenuated eastern equine encephalitis virus (EEEV), and attenuated Sindbis virus (SINV). In short, CHIKV structural proteins were engineered into the backbones of the aforementioned vaccines to produce the chimeras [3]. These chimeras were found to stimulate a strong humoral immunity, and even at doses of 5.3–5.8 log_10_ PFU, they did not trigger reactogenicity. When vaccinated mice were challenged with CHIKV, neither adult nor neonatal mice gained weight, had fever, or displayed signs of neurological illness. Upon comparison of the chimeras with the Army181/25 vaccine, the Army vaccine resulted in higher levels of viremia and replication in the joints of neonatal mice. Because the joints are known targets of CHIKV, Wang et al. noted their vaccine might avoid the negative reactogenic side effects of the Army vaccine. After being subcutaneously vaccinated with 5.3–5.8 log_10_ PFU of the chimeric vaccines, mice produced strong neutralizing antibody titers. The VEEV and EEEV chimeras yielded higher neutralizing antibody titers than the SINV chimera without being more virulent. On top of this, the VEEV and EEEV CHIKV chimeras seemed to be more immunogenic than the Army vaccine despite the chimeras' lower viremia and replication in the joints of neonatal mice [3].

Tiwari et al. [46] adopted a different strategy using formalin inactivated CHIKV in combination with alhydrogel (Aluminum Hydroxide) as an adjuvant. This study clearly suggests that this vaccine elicits both humoral and cell-mediated immune responses in mice, providing its immunogenic potential. A recent study by Couderc et al. [47] showed passive immunization as a potential treatment for CHIKV infection. Using purified immunoglobulin extracted from convalescent CHIKV patients, they demonstrated effective neutralizing activity against CHIKV infection both in vitro and in vivo. This thereby establishes a potential preventive and therapeutic approach to combat CHIKV infection. Pathogenesis studies conducted with related alpha virus, like RRV, have shown the role of macrophages in persistence on infection [48]. They also demonstrated the role of RRV-specific CD8 T cells in clearing viral load in infected patients, thereby warranting similar investigations with CHIKV and the importance of investigating a cell-mediated immune response-based vaccine against CHIKV [49].

There are always certain risks associated with live attenuated or inactivated viral vaccines [50]. One way to avoid these potential problems is to construct a consensus-based DNA vaccine. DNA based vaccines have an improved safety profile as compared to live or attenuated vaccines [51], [52]. A consequence of CHIKV's rapid evolution is difficulty in constructing a vaccine that will be able to effectively protect large populations from multiple strains of the virus. One of the strengths of DNA consensus vaccines is its ability to induce cross-reactive immune responses against the three distinct phylogenetic groups of CHIKV. Also DNA-based vaccines can be produced more rapidly than protein-based vaccines.

Recently, Muthumani et al. constructed a vaccine that was shown to induce both humoral and cellular immunity in vivo in 3–4-wk-old female C57/BL6 mice [49]. These mice were immunized using an in vivo electroporation method to deliver the vaccine into the quadriceps muscle. The consensus construct was designed against E1, E2, and the core protein capsid. To design the construct, they aligned 21 sequences of CHIKV isolated between 1952 and 2006, using strains from differing countries, including La Reunion Island. The most common nucleotide among the sequences was chosen at each position to be used in the consensus construct, taking care not to alter the reading frame. They conducted codon and RNA optimization, added a strong Kozak sequence, and substituted signal peptide with an immunoglobulin E leader sequence to improve vaccine efficacy.

**Figure 3:**
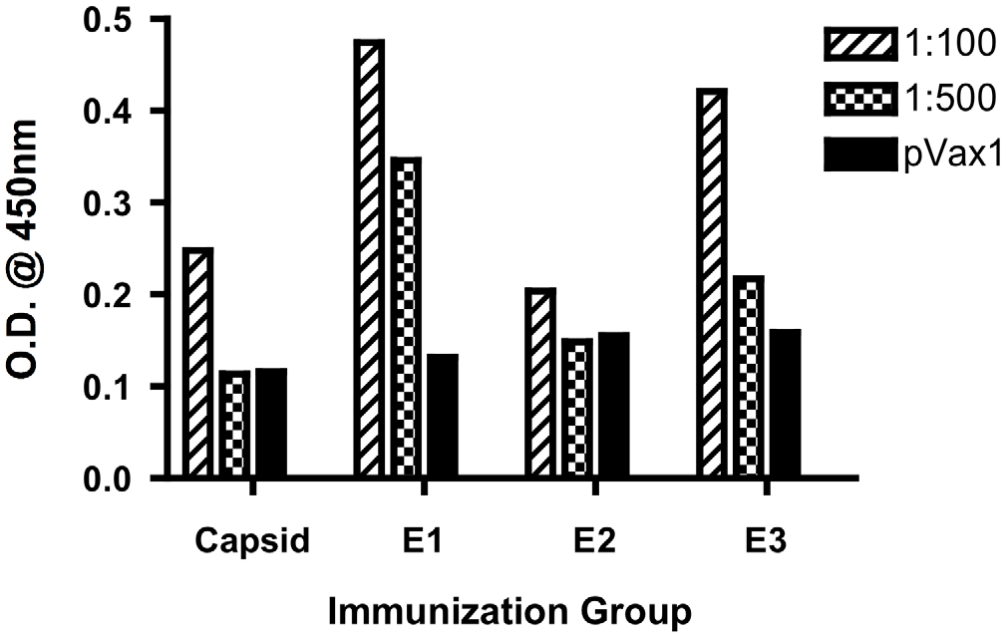
Levels of CHIKV-specific IgG in mice immunized with CHIKV vaccines. Each group of C57BL/6 mice (*n* = 5) was immunized with 12.5 µg of pVax1 control vector or CHIKV vaccine plasmids as indicated at 0 and 2 wk. Mice were bled 2 wk after each immunization, and each group's serum pool was diluted to 1∶100 and 1∶500 for reaction with specific vaccine constructs. Serum was incubated for 1 h at 37°C on 96-well plates coated with 2 mg/ml of respective CHIKV peptides, and antibody was detected using anti-mouse IgG-HRP and OD was measured at 405 nm.

**Figure 4:**
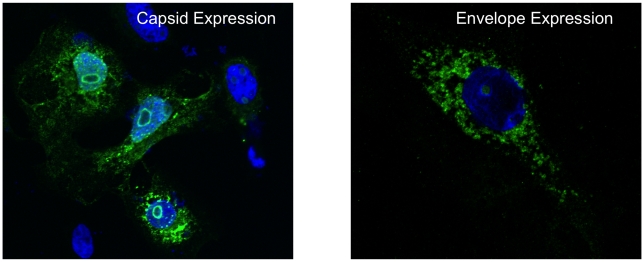
DNA vaccinated mice are capable of producing antibodies against the antigens encoded in the DNA vaccine. Hela cells transfected with DNA plasmid vaccine encoding the CHIKV Capsid (left) and Envelope (right) genes were examined for protein expression using confocal microscopy. Serum collected from mice immunized with the DNA vaccine was used as the primary antibody for detection of CHIKV proteins. Two days post-transfection, the cells, treated with serum and then with an anti-mouse IgG conjugated with Alexa-Fluor 488, were visualized under the Ziess LSM510 META NLO Laser Scanning Confocal Microscope (×63). Expression of high levels of CHIKV proteins in these cells revealed the presence of CHIKV-specific antibodies, thereby validating the efficacy of the DNA vaccine in inducing antibodies.

After immunizing the mice, spleens were harvested along with serum and tested to determine antibody titer. After three immunizations, consensus E1, E2, and C vaccines were shown to induce T-cell immune responses leading to strong IFN-γ responses and proliferation in C57/BL6 mice. Furthermore, when compared with control mice, immunized mice had higher total IgG levels as well as higher anti-E1 specific, anti-E2 specific, and anti-C specific IgG antibodies, suggesting a strong humoral immune response (Figure 3) and also specificity for the antigens encoded in the vaccine constructs (Figure 4). Because of its promising results and the need for a safer vaccine, this consensus DNA vaccine deserves further investigation. Determining longevity of protective effects of the vaccine and persistence of antibody and IFN-γ responses could be the next step of investigation. Challenged studies of immunized mice must also be carried out.

## Conclusion

CHIKV mosquito-borne disease has caused massive outbreaks for at least half a century but is no longer confined to the developing nations. It began to encroach into the boundaries of the developing world. As a result, the NIAID has designated CHIKV as a Category C pathogen alongside the influenza and SARS-CoV viruses [3]. Realization of the potential severity of this disease is exigent; for instance, if used as a biological weapon, the world economy could be severely crippled; if enough members of the armed forces were to become infected during a military deployment, military operations could be significantly affected. Efforts to monitor the disease will only provide minimal warning in a global society, and steps to prevent the morbidity and mortality associated with pandemic are imperative [21], [31].

Despite the gravity of its infectious potency and the fear of it being a potential biological weapon, there is currently no vaccine for CHIKV infections. Live attenuated vaccine trials were carried out in 2000, but funding for the project was discontinued. Newer approaches such as DNA vaccines appear promising over conventional strategies like live attenuated or inactivated virus and thus call for further investigation. Recent advances such electroporation delivery and incorporation of adjuvants has boosted DNA vaccine efficacy [51], [53]. Despite the low antibody response to DNA vaccines, other numerous advantages have overshadowed these minor drawbacks (Table 2), the most important one being the ability to induce both humoral and cellular immune responses [51], [54].

**Table 2:** Comparative properties of DNA vaccines over other vaccine approaches.

Property	Live Attenuated Virus	Killed Viral Particle	DNA Vaccine	Reference
Manufacture & design	Laborious design process	Simpler process but requires meticulous monitoring of process parameters to conserve potency	Simple molecular genetic processes involved in manufacturing and plasmid optimization	[52]
Cell-mediated responses	Good	Poor	High	[53], [64], [65]
Antibody responses	Mainly IgG	IgA and IgG	No significant antibody response	[53]
Safety	Possible reversion to virulence	Lesser likelihood of reversion	No possibility of virulence or toxicity reported	[52], [66]
Duration of immunity	Many years	Lesser duration	Long-term immunogen persistence	[52], [54], [65]
Post-manufacturing stability	Requires continuous cold chain sustenance	Requires preserving in cold chain as it is heat liable	Does not require cold chain and has better shelf-life	[52], [66]

Judging by recent success, such as the immunogenic construct developed by Muthumani et al., DNA vaccines could play a major role in combating CHIKV [49]. Vaccines are literally a critical component of CHIKV disease control and therefore research in this area is highly encouraged. The dramatic spread of dengue viruses (DENV) throughout tropical America since 1980 via the same vectors and human hosts underscores the risk to public health in the Americas. The adverse events associated with the current live vaccine are well documented [55]. Realizing these drawbacks, earnest efforts should be taken to develop new strategies to forestall further spread and complications.

**Key Learning Points**

- Chikungunya biology and possible global expansion
- Pathogenesis and clinical diagnostic aspects of CHIKV
- Common symptoms and complications involved in CHIKF
- Current status of anti-CHIKV therapies
- Finally nominates a novel envelope-based DNA vaccine approach against CHIKV infection

**Key References**

1. Vazeille M, Moutailler S, Coudrier D, Rousseaux C, Khun H, et al. (2007) Two Chikungunya isolates from the outbreak of La Reunion (Indian Ocean) exhibit different patterns of infection in the mosquito, Aedes albopictus. PLoS ONE 2: e1168.
2. Tsetsarkin KA, Vanlandingham DL, McGee CE, Higgs S (2007). A single mutation in chikungunya virus affects vector specificity and epidemic potential. PLoS Pathog 3: e201.
3. Queyriaux B, Simon F, Grandadam M, Michel R, Tolou H, Boutin, J.P (2008). Clinical burden of chikungunya virus infection. Lancet Infect Dis 8: 2–3.
4. Muthumani K, Lankaraman KM, Laddy DJ, Sundaram SG, Chung CW, et al. (2008). Immunogenicity of novel consensus-based DNA vaccines against Chikungunya virus. Vaccine 26: 5128–5134.
5. Ng LF, Chow A, Sun YJ, Kwek DJ, Lim PL, et al. (2009). IL-1beta, IL-6, and RANTES as biomarkers of Chikungunya severity. PLoS ONE 4: e4261.
